# Arsenic Exposure Causes Global Changes in the Metalloproteome of *Escherichia coli*

**DOI:** 10.3390/microorganisms11020382

**Published:** 2023-02-02

**Authors:** James Larson, Monika Tokmina-Lukaszewska, Hunter Fausset, Scott Spurzem, Savannah Cox, Gwendolyn Cooper, Valérie Copié, Brian Bothner

**Affiliations:** Department of Chemistry and Biochemistry, Montana State University, Bozeman, MT 59715, USA

**Keywords:** arsenic, iron, zinc, copper, magnesium, *Escherichia coli*, metalloproteome, inductively coupled plasma mass spectrometry

## Abstract

Arsenic is a toxic metalloid with differential biological effects, depending on speciation and concentration. Trivalent arsenic (arsenite, As^III^) is more toxic at lower concentrations than the pentavalent form (arsenate, As^V^). In *E. coli*, the proteins encoded by the *arsRBC* operon are the major arsenic detoxification mechanism. Our previous transcriptional analyses indicate broad changes in metal uptake and regulation upon arsenic exposure. Currently, it is not known how arsenic exposure impacts the cellular distribution of other metals. This study examines the metalloproteome of *E. coli* strains with and without the *arsRBC* operon in response to sublethal doses of As^III^ and As^V^. Size exclusion chromatography coupled with inductively coupled plasma mass spectrometry (SEC-ICPMS) was used to investigate the distribution of five metals (^56^Fe, ^24^Mg, ^66^Zn, ^75^As, and ^63^Cu) in proteins and protein complexes under native conditions. Parallel analysis by SEC-UV-Vis spectroscopy monitored the presence of protein cofactors. Together, these data reveal global changes in the metalloproteome, proteome, protein cofactors, and soluble intracellular metal pools in response to arsenic stress in *E. coli.* This work brings to light one outcome of metal exposure and suggests that metal toxicity on the cellular level arises from direct and indirect effects.

## 1. Introduction

Arsenic is a well-known carcinogen and is one of the most prevalent environmental toxins [1,2,3]. In nature, the two main forms of inorganic arsenic are the trivalent form arsenite (As^III^) and the pentavalent form arsenate (As^V^), the former being more toxic [4]. Both arsenic species enter the cell through adventitious mechanisms. As^V^ is structurally similar to phosphate and is uptaken by the phosphate transporters, Pst and Pit. As^III^ enters the cell through the glycerol facilitator membrane protein GstF [5,6,7].

Because arsenic is readily assimilated, organisms have evolved to detoxify arsenic. In bacteria, this is primarily accomplished by proteins encoded by *ars* genes. There are many forms of the *ars* operon, but in its simplest form, only three proteins are encoded. This is known as the *arsRBC* locus. In a counterintuitive approach, As^V^ must first be reduced to the more toxic As^III^ by ArsC, and is then removed through the efflux pump ArsB (or ArsAB). ArsR is a DNA-binding regulator protein that controls the expression of the other *ars* operon proteins [7,8,9,10]. Previous studies of the bacterium *Agrobacterium tumefaciens* 5A have shown that an intertwined genetic circuit exists between phosphorous uptake genes and arsenic detoxification genes [11,12,13]. A more recent study has shown that the ArsR protein also affects metabolism [9]. Using RNAseq, this study found that the ArsR proteins of *Agrobacterium tumefaciens* 5A also influence many biological processes, including sugar transport, chemotaxis, copper tolerance, and iron homeostasis.

Organisms use metalloproteins to achieve distinct and specific biologic functions. As a consequence of this, metal ions are crucial in biology, and organisms have evolved mechanisms to maintain metal homeostasis [14]. Imbalances in this homeostasis have effects on other metals, protein expression, and cofactor abundance. For example, high levels of zinc in *E. coli* lead to an increased demand for iron, along with decreased copper levels [15]. In *Caenorhabditis elegans*, exposure to Mn alters the Ca and Fe balances, as well as protein homeostasis [16]. To date, there are limited studies of how arsenic affects other metals. Unraveling the interplay between arsenic and other metals will provide a deeper understanding of arsenic toxicity.

In the present study, an *E. coli* strain containing the *arsRBC* operon K-12 and a strain lacking the operon AW3110 were cultured in sublethal doses of trivalent arsenic As^III^ or pentavalent arsenic As^V^. Using inductively coupled plasma mass spectrometry (ICP-MS), size-exclusion chromatography coupled to ICP-MS (SEC-ICPMS), and SEC coupled to a diode array UV detector (SEC-UV), changes were detected in the metalloproteome of five metals (^75^As, ^56^Fe, ^24^Mg, ^66^Zn, and ^63^Cu). The observed changes were different between the two strains of *E. coli* and depended on treatment with trivalent or pentavalent arsenic. Our findings extend understanding of the intricate biological coordination underlying arsenic regulation while opening a window for understanding the interplay between metal balances and cellular metabolism.

## 2. Materials and Methods

### 2.1. E. coli Cell Growths

*E. coli* K12 cultures were grown in 200 mL of LB broth as batch cultures at 37 °C. Cultures were inoculated from an overnight culture that was started from a single colony selected from an LB agar plate. A total of 2 h after inoculation (early log phase), cells were treated with 1 mM As^III^, 1.5 mM As^V^, or left untreated. Batch cultures to establish arsenic concentrations and timing for the experiment were repeated over the span of months. For the cultures used for analysis, each condition had three biological replicates. Replicates for a given condition were grown on the same day. Cells were pelleted via centrifugation at 4000 x g for 10 min, 4 h post-arsenic administration. Cell pellets were carefully washed with 1 mM EDTA in PBS. A final centrifugation step was performed to harvest the cells. The cells were stored at −80 °C until further analysis. *E. coli* AW3110 cultures were grown under chloramphenicol selection in 200 mL of LB broth at 37 °C in the same fashion as for K-12. At 2 h post-inoculation (early log phase), 100 µM As^III^ or 500 µM As^V^ was administered to the test cultures, while the control culture was left untreated. The As^V^ condition was grown in quadruplicate, while the control and As^III^ conditions were grown in triplicate. At 4 h after arsenic administration, or 6 h after inoculation for control cultures, cells were pelleted, washed, harvested, and stored in the same manner as the K12 cultures. The K12 and AW3110 cell lines were a gift from Dr. Barry Rosen and are as described in the original publication [17].

### 2.2. Protein Extraction

Cell pellets were resuspended in 1.5 mL of 200 mM ammonium acetate pH 7 in the presence of the protease inhibitors (VWR) and immediately lysed using matrix E (MPBio Irvine, California, USA) and FastPrep-24 5G bead beater (MPBio Irvine, California, USA). Cells were then lysed with cycles at a speed of 6.0 m/s for 30 s on a FastPrep-24 5G bead beater (MPbio Irvine, California, USA). The cell debris and the matrix were pelleted at 18,000× *g* for 20 min and the soluble fraction was collected. Protein concentration was determined using a Bradford assay.

### 2.3. Metalloproteome Analysis

The metalloproteomes for each condition were analyzed via SEC-ICP-MS using an Agilent Infinity II LC coupled to an Agilent 7800 ICP-MS. The methods were inspired by work done by the Roberts lab [18,19]. A total of 160 µg of soluble protein (entire proteome) was injected onto an Agilent Bio-SEC 3 (3 µm, 300 Å, 4.6 × 300 mm) column and proteins were separated for 25 min using 200 mM ammonium acetate pH 8 as the mobile phase, with a flow rate of 0.4 mL/min. The five metal ion signals were monitored, and these included: ^24^Mg, ^56^Fe, ^63^Cu, ^66^Zn, and ^75^As, with an integration time/mass of 1.5 s per analyte. The monitoring of ion signals was performed using the Agilent MassHunter 4.6 (version C.01.06). Metal signals were manually integrated in MassHunter, and all other data workup was performed in Microsoft Excel. Statistical differences were determined using a Student’s t test at a 95% confidence interval.

### 2.4. Total Metal Analysis of Soluble Fraction Digest and Media

Biologic replicates were diluted to the same protein concentration. Then equal parts were pooled to create one sample per condition. The metals were liberated from biologic molecules using a 5% nitric acid digestion. The precipitate was pelleted via centrifugation, and the supernatant was collected for analysis. Metal analysis was performed using an Agilent Infinity II autosampler coupled to an Agilent 7800 ICP-MS. The mobile phase was 2% HNO_3_, 0.5% HCl in water, with a flow rate of 1 mL/min. The signals of five metal ions were monitored for 30 s, and included: ^24^Mg, ^56^Fe, ^63^Cu, ^66^Zn, and ^75^As, with an integration time/mass of 0.5 s per analyte. The quantification of analytes was performed in Agilent MassHunter 4.6 (version C.01.06) against a standard curve. Media with and without arsenic was digested in the same fashion The metals of interest were analyzed using an Agilent SPS 4 autosampler coupled to an Agilent 7800 ICP-MS in helium mode. The quantification of analytes was performed against a standard curve in MassHunter.

### 2.5. ICP-MS Instrument Parameters

For small volume LC analysis (Agilent Infinity II coupled to Agilent 7800 ICP-MS), the following instrument parameters were used: RF power 1550 V, RF matching 1.00 V, nebulizer gas 0.99 L/min, option gas 0.0%, nebulizer pump 0.30 rps, S/C temp 2 °C, makeup gas 0.00 L/min, extract 1 lens 0.0 V, extract 2 lens −195.0 V, omega bias −95 V, omega lens 8.4 V, cell entrance −40 V, cell exit −60 V, deflect 0 V, plate bias −55 V, He flow rate 4.0 mL/min, octupole bias −18 V, octupole RF 200 V, and energy discrimination 3.0 V.

For large volume analysis (Agilent SPS 4 coupled to Agilent 7800 ICP-MS), the following instrument parameters were used: RF power 1550, RF matching 1 V, nebulizer gas 1.00 L/min, option gas, 0.0 %, nebulizer pump 0.10 rps, S/C temp 2 °C, makeup gas 0.00 L/min, extract 1 lens 0.0 V, extract 2 lens −200.0 V, omega bias −105 V, omega lens 7.5 V, cell entrance −40, cell exit −60 V, deflect −0.6 V, plate bias −55 V, He flow rate 4.5 mL/min, octupole bias −18.0 V, octupole RF 200 V, and energy discrimination 5.0 V.

### 2.6. Proteome Analysis

The native-state soluble proteome was fractionated by collecting samples from the Bio-SEC 3 column with the same method used for ICP-MS, except using a different LC system (Agilent 1290 Infinity) with an Agilent Infinity diode array detector. A total of 200 µg of total protein was manually injected, and tube lengths were matched to ensure profiles were comparable with the SEC-ICPMS profiles. Starting at 4 min post-injection, 1 min fractions were collected manually for a total of 10 min. Fractions were freeze-dried for subsequent SDS-PAGE analysis. The freeze-dried samples were resuspended in a 240 mM Tris HCl pH 6.8 buffer containing 277 mM SDS and 40% SDS, heated at 99 °C for 6 min, and loaded onto a 4–20% Mini-Protean ^®^ TGX ™ Precast 15 µL-well (Bio-Rad Hercules, California, USA). Gels were stained with GelCode ™ Blue Stain Reagent (ThermoFisher Waltham, Massachusetts, USA).

## 3. Results

### 3.1. Arsenic Induced Changes in the Metalloproteome of E. coli K-12

**Figure 1:**
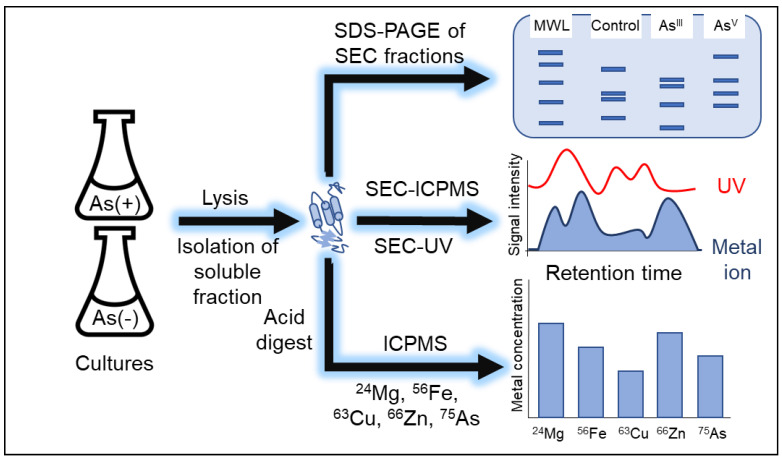
Metalloproteomics workflow. Replicate cultures of K12 and AW3110 +/− As were cultured. Cells were lysed, and the soluble fraction was collected. Size-exclusion chromatography (SEC) was used for fractionating the native proteome prior to analysis by SDS-PAGE. SEC was used in-line with inductively coupled plasma mass spectrometry (SEC-ICPMS) for analysis of the native metalloproteome and in-line with a diode array detector for analysis of UV active cofactors (SEC-UV). The soluble fractions were digested with nitric acid for the analysis of cellular metal content via inductively coupled plasma mass spectrometry (ICP-MS).

*E.* coli strains K-12 and AW3110 were selected for this study because AW3110 lacks the *arsRBC* operon, while K-12 has an intact *arsRBC* operon [17]. A metalloproteomics workflow was developed for this study (Figure 1). Batch cultures were grown in sublethal concentrations of As^III^ or As^V^ (Supplemental Figure S1). After 4 h of exposure to arsenic, the soluble fraction of the proteome was extracted under native conditions to keep protein complexes and protein-metal interactions intact. The metalloproteomes from different conditions were analyzed by size exclusion chromatography coupled with inductively coupled plasma mass spectrometry (SEC-ICPMS), while acid digestion following ICP-MS was used to quantify total cellular metals. SEC coupled to a UV-Vis diode array detector (SEC-UV) was used to monitor the wavelengths of the protein-bound cofactors and to fractionate the soluble proteome. Denaturing SDS-PAGE was used to visualize the soluble proteome and the proteins in each SEC fraction. Together, these techniques provide insight into the changes induced by As^III^ and As^V^ treatments in *E. coli* K-12 and AW3110.

The isolated soluble native protein fractions of *E. coli* K-12 untreated, As^III^ exposed, and As^V^ exposed were analyzed using SEC-ICPMS to determine the effects of As^III^ and As^V^ on the metalloproteome of *E. coli*. Specifically, ^56^Fe, ^24^Mg, ^66^Zn, and ^63^Cu ions were monitored, as proteins containing these metals are involved in DNA/RNA metabolism, oxidation/reduction, and other critical biological functions, while ^75^As was monitored to detect the integration of arsenic into the proteins. SEC-ICPMS was used under non-denaturing conditions to investigate the native proteome and maintain metal–protein interactions. 

**Figure 2:**
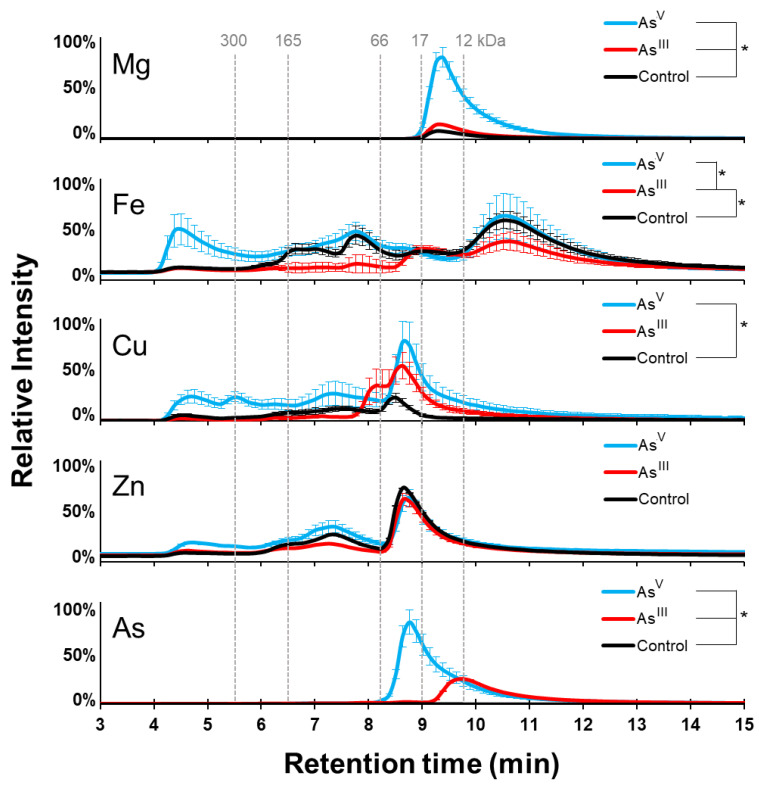
K-12 metalloproteome response to arsenic treatment. SEC-ICP-MS analysis shows the size distribution of protein-bound copper, magnesium, arsenic, iron, and zinc. As^V^ treatment is shown in cyan, the As^III^ treatment in red, and the control in black. Error bars are +/−1 standard deviation. * Indicates significant differences (*p* < 0.05) determined by a Student’s *t*-test (n = 3).

As expected, there was a negligible arsenic signal for the control sample, while both arsenic treatments showed arsenic inclusion into the proteome (Figure 2). The arsenic-containing protein peak observed in the As^V^ treatment peaked slightly before minute 9 on the SEC, while the As^III^ treatment peak occurred slightly before minute 10. A Student’s *t*-test (*n* = 3) showed a significant increase (*p*-value = 0.011) in arsenic in the soluble fraction from the As^V^ treatment compared to the As^III^ treatment group (Supplemental Figure S2). These results show that cells treated with arsenic incorporate the toxic metal into their cellular proteome.

We also investigated the effect of arsenic treatment on the magnesium proteome. There was only one elution peak for magnesium-containing proteins, and it showed a maximum signal between minutes 9 and 10 in every condition tested (Figure 2). This peak was significantly increased (*p*-value = 0.012) in the As^III^ treatment compared to the control group, and was further increased with As^V^ treatment (*p*-value = 0.00087). This result shows that arsenic treatments increase the abundance of magnesium or magnesium-containing proteins, specifically in the case of As^V^ treatment.

The integrated iron signal of the proteome was significantly higher (*p*-value = 0.040) in the control cell cultures compared to the As^III^ treated groups, and the iron signal was significantly higher in the As^V^ treatment than the As^III^ treatment group (*p*-value = 0.039) (Figure 2). Total iron was not significantly different between the As^V^ treatment and the control; however, the distribution of iron throughout the native proteome showed noticeable changes between all three groups. The maximum in the iron signal was observed in all three conditions between minutes 10 and 11. The maximum was near the same intensity in both the control and As^V^ treated cell cultures, with a decreased signal in the cells treated with As^III^. An increase in iron signal intensity was observed between minutes 4 and 5 in the As^V^ group, with only a minor signal observed in the control and As^III^ treated groups. A decrease in the iron signal between minutes 6 and 8.5 was present in the As^III^ treatment group as well. Together, the data suggest that arsenic treatment changes the iron proteome of *E. coli* K12 cells, with the greatest effects being noted in proteins and protein complexes greater than 66 kDa in size.

We next examined the effects of arsenic treatment on copper-containing proteins and zinc-containing proteins. As^V^ treatment significantly increased copper (*p*-value = 0.031) across the entire elution profile compared to the control group (Figure 2). In contrast, As^III^ treatment caused an increase in the copper signal between minutes 8 and 10; however, the abundance of copper in the proteome was not significantly different. These results suggest that As^V^ treatment has a larger effect on the copper proteome than does the As^III^ treatment, which increases the abundance of copper-containing proteins that are ~66 kDa in size. The zinc signal under all conditions was at a maximum near minute 9. This peak was indistinguishable between conditions, and no significant differences (*p*-value < 0.05) in the zinc-containing proteins were observed between conditions. This data indicates that in K-12, the zinc proteome is unchanged following arsenic treatments.

### 3.2. Arsenic Induced Changes in the Metalloproteome of E. coli AW3110

*E. coli* AW3110 lacks the genes primarily responsible for the cellular detoxification of arsenic. We used this strain to address how the metalloproteome changes in response to arsenic when the *arsRBC* operon is absent, in theory increasing the arsenic available to the proteome soluble protein fractions of *E. coli* AW3110. The concentrations of As^III^ and As^V^ were chosen to elicit similar levels of arsenic stress as K-12. The AW3110 control, As^III^, and As^V^ exposed cultures were analyzed with SEC-ICPMS, as performed above for the *E. coli* K-12 strain.

**Figure 3:**
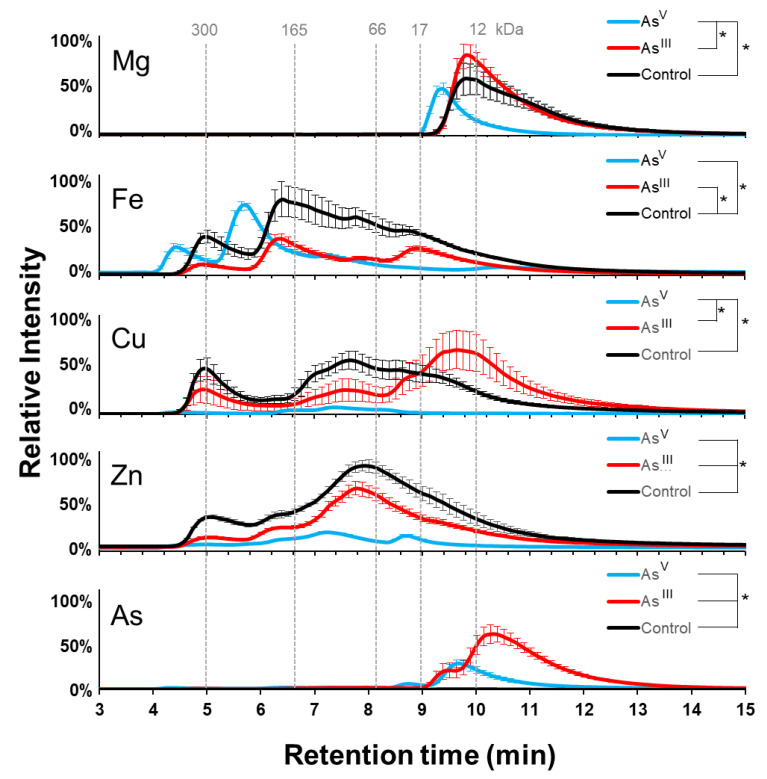
AW3110 Metalloproteome response to arsenic treatment: Metal profiles for copper, magnesium, arsenic, iron, and zinc from SEC-ICP-MS analysis. As^V^ treatment is shown in cyan, As^III^ treatment is in red, and control is in black. Error bars are +/−1 standard deviation. * Indicates significant differences (*p* < 0.05) determined by a Student’s *t*-test (n = 3 for As^III^ and control; n = 4 for A^V^ treatment).

Treatment with both species of arsenic resulted in arsenic incorporation into the soluble proteome. The As^III^ condition showed a signal between minutes 9 and 13, with the highest intensity between minutes 10 and 11 and a shoulder between minutes 9 and 10 (Figure 3). As^V^ treatment, however, showed significantly fewer (*p*-value < 0.0016) arsenic-containing proteins, which eluted primarily between minutes 9 and 10, and a smaller maximum peak between minutes 8 and 9. As expected, there was a negligible arsenic signal in the proteins from the control group. These data are consistent with the arsenic incorporation into the proteome of *E. coli* K-12 cells.

The profiles of the magnesium-containing protein appeared to be very similar between the control and As^III^ treated groups, while the As^V^ treatment caused a slight shift towards earlier eluting proteins (Figure 3). As^V^ treatment also caused a significant decrease (*p*-value = 0.0098) in the total amount of magnesium-bound protein and a decrease in total soluble magnesium (Supplemental Figure S3). The iron signals showed that the As^III^ treatment and the control were very similar, except for a reduced signal intensity in the As^III^ treatment (*p*-value < 0.0028). The As^V^ treatment group yielded two strong iron signals, with maxima between minutes 4 and 5 and between minutes 5 and 6. Proteins and protein complexes eluting after six minutes displayed a diminished signal for iron compared to that in the control group. Despite the different elution profiles, there was no significant difference in total iron signal between As^V^ and As^III^ treatments (*p*-value = 0.51). These results indicate that arsenic imparts changes in the AW3110 magnesium metalloproteome that are distinct from those observed for *E. coli* K-12.

The effect arsenic treatment had on copper-containing proteins showed that the As^V^ treatment significantly diminished most of the copper-containing proteins (*p*-value = 0.0042) (Figure 3). The copper signal observed in the control at minute 5 and between minutes 7 and 8 were decreased in the As^III^ treated sample. As^III^ treatment also yielded lower molecular weight copper-containing proteins. As^III^ treatment significantly reduced the zinc-containing proteins compared to the control sample, but maintained a similar elution profile (*p*-value = 0.0062). As^V^ treatment resulted in a significantly decreased zinc signal compared to the As^III^ (*p*-value = 0.000083) and control (*p*-value = 0.000022) conditions. It is clear that arsenic treatment has dramatic effects on the copper proteome, and that both arsenic treatments reduce the abundance of zinc or zinc-containing proteins across the entire elution profile, with As^V^ having the largest effect.

### 3.3. Changes in Total Cellular Metal

**Figure 4:**
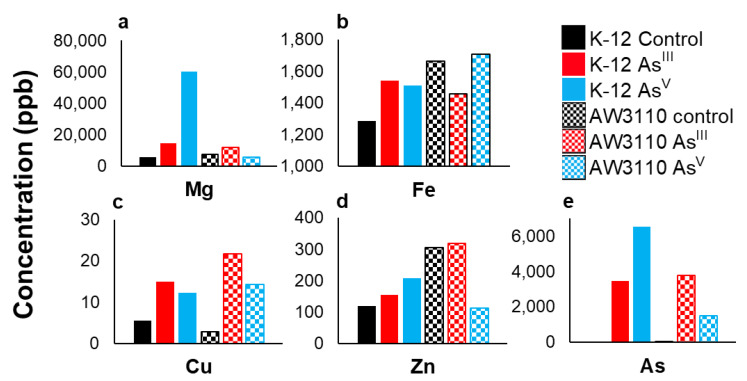
Total intracellular soluble metal. Pooled soluble fractions for K-12 (solid fill) and AW3110 (checkerboard) growths analyzed by ICP-MS for magnesium (**a**), iron (**b**), copper (**c**), zinc (**d**), and arsenic (**e**). As^V^ treatment is shown in cyan, As^III^ treatment is in red, and control is in black. The concentration is given above each sample in ppb.

SEC-ICPMS experiments provide information about the metalloproteome, but provide no information about metal intake inside the cell. To establish this, we determined the total cellular pool of the metal in each group. To measure the total soluble metal pools of each treatment group, the metals were liberated from biomolecules using a nitric acid digestion. Biological replicates were analyzed by ICPMS for magnesium, zinc, iron, arsenic, and copper (Figure 4). The pooled K-12 control sample had a 5.7 ppm magnesium concentration. Arsenic treatment caused a 2.5-fold and a 10.5-fold increase in magnesium concentration relative to the control for the As^III^ and As^V^ treatments, respectively. In AW3110, the pooled control sample had a magnesium concentration of 7.2 ppm. There was an increase in the As^III^ treatment samples and a slight decrease for the As^V^ treatment samples (Figure 4a). Iron concentration increased slightly from 1.29 ppm in the As^III^ and As^V^ conditions (Figure 4b). Cellular copper levels rose with both arsenic treatments, with the largest increase seen in the As^III^ treatment for both cell strains (Figure 4c). In *E. coli* K-12, the As^III^ treatment was associated with an increase in zinc concentration in both treatment groups. In the *E. coli* AW3110 strain, the control and As^III^ treatment zinc concentrations were 304 and 317 ppb, respectively, while the As^V^ treatment reduced the zinc concentration to approximately one-third of this value (Figure 4d). Less than 5 ppb of arsenic was observed in the control samples of both cell lines. As^III^ treatment resulted in 3.47 ppm and 3.77 ppm arsenic concentrations for the K-12 and AW3110 samples, respectively. As^V^ treatment led to an arsenic concentration of 6.5 ppm in the *E. coli* K-12 and 1.5 ppm in the AW3110 cells (Figure 4e). Media with and without As^III^ or As^V^ was analyzed by ICP-MS to check for background arsenic in the stock solutions (Supplemental Figure S4). These results demonstrate that arsenic treatment not only changes the concentration and abundance of metal-bound proteins, but also alters the cellular metal concentration.

### 3.4. Changes in the Soluble Proteome

In addition to changes in metal abundance, the SEC-ICPMS profile of the metalloproteome can be affected by differences in the abundance of proteins and the presence of protein complexes. To determine if and how the soluble proteome changed, an SDS-PAGE analysis was conducted on the total soluble proteome of the collected SEC fractions for the control, As^III^, and As^V^ treated cells for both K-12 and AW3110 *E. coli* strains. SDS-PAGE analysis is performed under denaturing conditions breaking about protein complexes. Differences in the soluble proteome were observed between all conditions for K-12 and AW3110 (Supplemental Figure S5). Gel lanes corresponding to the SEC fractions made those differences more apparent. In the soluble protein fractions of K-12, a protein band near 30 kDa was present in the As^V^ conditions, but absent in the control and As^III^ conditions (Supplemental Figure S5a). Similarly, there was a protein band near 50 kDa that was present in fractions 4–7, which was absent or minimally present in the arsenic conditions. The banding patterns in the As^III^ and As^V^ conditions between 37 and 50 kDa were also different in fraction 4. In the soluble protein fractions of AW3110, a protein band near 50 ka was present in fraction 2 of the control and As^V^ treated cells, but was minimally present in the cells treated with As^III^ (Supplemental Figure S5b). In fraction 3, there was a protein band near 50 kDa that was present in the As^III^ and As^V^ treated groups, but was more abundant in the control group. The protein bands between 10 and 15 kDa in fraction 3 were nearly absent in the As^V^ treated cells, but were present in the control and As^III^ treated cells. The SDS-PAGE analysis indicated significant differences in the soluble proteome profiles across all conditions.

### 3.5. Protein Cofactor Changes

**Figure 5:**
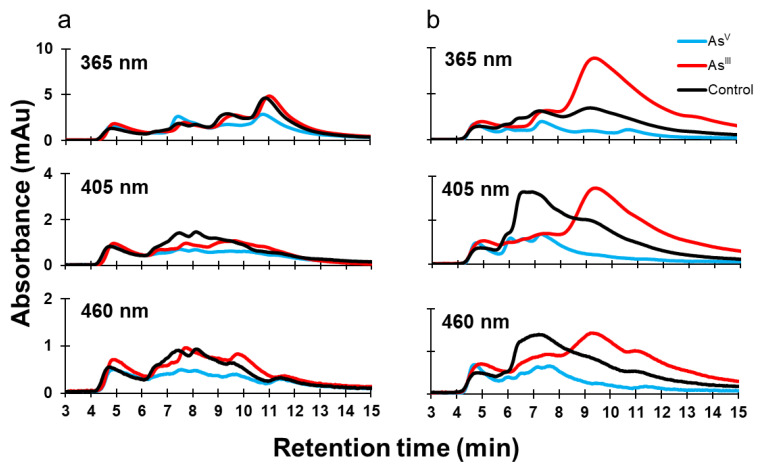
Protein-bound cofactor distribution changes caused by arsenic treatment. Soluble proteomes were analyzed on an SEC coupled with a DAD detector. The wavelengths 365, 405, and 460 nm were monitored for K-12 (**a**) and AW3110 (**b**) under three conditions: control (black), As^V^ (cyan), and As^III^ (red).

Organisms use enzymes to achieve specific cellular outcomes, and enzymes often require cofactors to function properly. Because of this, environmental conditions (such as the presence of heavy metals) will influence protein expression and cofactor abundance, as well as the redox status of cells. Many biological cofactors absorb in the UV and visible spectrum, and often, the cofactor will change its spectroscopic properties depending on its redox status. For example, oxidized flavin has two absorption peaks near 365 nm and 460 nm, while reduced flavin becomes spectroscopically inactive at these wavelengths [20]. Another important cofactor is heme, which absorbs at 405 nm [21]. Samples that were analyzed by SEC-ICPMS were pooled for each condition and analyzed by SEC-UV to determine whether there was a change in the detectable differences of protein-bound cofactors (Figure 5). In K-12 cells, As^V^ treatment decreases in absorbance at 365 nm between minutes 9 and 13, at 405 nm between minutes 6 and 12, and at 460 nm between minutes 6 and 11. As^III^ treatment of K-12 resulted in very similar UV signal at 365 nm, but a decreased signal at 450 nm between minutes 6 and 10. The signal profiles are different for 405 nm and 460 nm (Figure 5a). The growth of AW3110 in the presence of As^III^ caused increases in the 365 nm signal between minutes 8 and 15, and in the 405 nm and 460 nm signals between minutes 8.5 and 15, while decreases in the 405 nm and 460 nm signals were observed between minutes 6 and 8.5 (Figure 5b). As^V^ treatment caused decreases in the 365, 405, and the 460 nm signals between minutes 5.5 and 15. Our data suggest that the cofactor distribution and abundance in the proteome of AW3110 is more susceptible to changes induced by arsenic treatments, with As^III^ having a more profound effect than As^V^ and coinciding with Cu and Mg metal distributions.

## 4. Discussion

*E. coli* strains K-12 and AW3110 were given sub-lethal doses of the most prevalent arsenic toxins, arsenite (As^III^) and arsenate (As^V^). K-12 has an intact arsenic detoxification mechanism that is encoded by the *arsRBC* genes. AW3110 lacks these genes and is more susceptible to arsenic toxicity. Little is known about how arsenic affects the assimilation of other metals, or how the arsenic detoxification machinery is involved with metal perturbations inside the cells. Our experiments shed new light on the perturbations of cellular metal homeostasis caused by arsenic.

The foundation for our work came from the transcriptomic study of Rawle et al. on *Agrobacterium tumefaciens* 5A. This bacterium has been used as a model organism for studying arsenic detoxification [9,12,13,22,23,24]. The genome encodes two *ars* loci, and each locus contains the basic components of arsenic detoxification: a reductase, an efflux pump, and repressor proteins, along with other proteins not discussed here. In total, *A. tumefaciens* has four arsR protein-encoding genes; *arsR1*, *arsR2*, *arsR3*, and *arsR4*. Rawle’s study knocked out each *arsR* gene individually prior to transcriptomic analysis. Their work showed that the *arsR* genes were involved in nearly every aspect of gene regulation and cell physiology when *A. tumefaciens* 5A was exposed to As^III^ [13]. This involvement includes both the repression and activation of metal-containing proteins. Their data revealed that genes coding for arsenic, copper, zinc, and iron-dependent proteins were both repressed and/or activated when As^III^ was introduced. Copper metabolism and iron transport/regulation genes were primarily downregulated with the highest As^III^ levels tested. While our current study lacks transcriptomic analyses, we see clear changes in the metalloproteomes of both *E. coli* strains upon arsenic treatments, except for the zinc metalloproteome of *E. coli* K-12 cells.

### 4.1. Arsenic

It has been demonstrated previously that cytosolic proteins isolated from rabbit liver incubated with As^III^ bound 13 times more arsenic than proteins incubated with As^V^ [25]. In K-12 cells, we see a noticeably higher arsenic protein signal for As^V^ treatment compared to As^III^ treatment, whereas with *E. coli* AW3110, the converse is true. K-12 encodes for the As^V^ reductase ArsC, which converts As^V^ into As^III^. K-12 cells were given 50% more arsenic in the As^V^ treatment than was provided during the As^III^ treatment. This may explain why there is more arsenic incorporation in K-12 than in AW3110 *E. coli* cells. Interestingly, there is a trend in both K-12 and AW3110, where As^V^ treatment incorporates arsenic into proteins with higher molecular weights (or complexes). While the expression of ArsC and a higher concentration of arsenic in the As^V^ treatment compared to the As^III^ treatment may explain increased arsenic incorporation in proteins, the observed trend of arsenic incorporation into higher molecular weight proteins in As^V^ treatment compared to As^III^ treatment in both cell strains remains enigmatic. Our data clearly show that arsenic incorporates into the proteome differently depending on the arsenic species provided to the cells, regardless of whether an arsenic detoxification mechanism is present or not.

### 4.2. Copper

Copper is coordinated by proteins, primarily through histidine, methionine, and cysteine side chains [26]. As^III^ may bind to the sulfhydryl groups of cysteines and displace liganded copper. A 2004 study looked at trivalent arsenicals binding to rat hemoglobin [27]. The research found that trivalent arsenicals bind only at cysteine residues, and that less than 10% of inorganic As^III^ is bound to rat hemoglobin, while monomethylarsonous acid and dimethylarsonous acid were bound to nearly 50% and 80%, respectively. This may explain why As^III^ treatment of AW3110 does not drastically reduce the proteome profile of copper-containing proteins. Rawle et al. saw both upregulation and downregulation by the *arsR* genes of transcripts encoding copper-containing proteins involved in copper transport, electron-transport, and copper detoxification in As^III^ treated cells [13]. It is plausible that this transcriptome pattern is present in As^V^ treated *A. tumefaciens* as well. In As^V^ treated K-12 cells, an increase in higher molecular weight copper-containing proteins was observed. In AW3110, however, As^V^ treatment drastically reduced the amount of copper-containing proteins. Interestingly, As^III^ treatment in AW3110 resulted in similar concentrations of copper-containing proteins, but with a different elution profile. 

### 4.3. Magnesium

One of the most surprising results included the large increase in the magnesium bound proteins when K-12 *E. coli* cells were treated with As^V^ (Figure 2), as well a large increase the total intracellular soluble magnesium intake (Figure 4a). Magnesium influx has been reported as a response to ribosomal stress in *Bacillus subtilitis*, which prevents the hyperpolarization of the membrane potential [28]. Furthermore, As^V^ has been shown to degrade ribosomes in *E. coli*, releasing phosphate into the cell [29]. This was demonstrated by growing *E. coli* in the presence of [^3^H]-uridine. The authors analyzed acid-soluble material released following ribosome degradation using thin-layer chromatography (TLC) and observed that As^V^-induced ribosomal degradation led to increased radioactivity in the TLC chromatogram, which corresponded to uracil, but not to UTP, UDP, or UMP. A different study using untargeted proteomic analysis revealed a downregulation of ribosomal protein-encoding genes in As^III^ treated yeast [30]. This was proposed to reflect a protection mechanism, rather than a toxic effect from As^III^. In the transcriptomic analysis by Rawle et al., only three magnesium related proteins were shown to be affected by As^III^ treatment. The three proteins were a putative adenylate cyclase, GroES, and a nudix hydrolase. Adenylate cyclases convert ATP into cAMP [31,32]. GroES is part of the GroEL-GroES chaperonin machine which provides a nano-cage for the correct folding of proteins [33]. Nudix hydrolases are a large class of enzymes which catalyze the hydrolysis of nucleoside diphosphates [34,35]. Our data, in conjunction with current literature, suggest that the presence of the *arsRBC* operon is likely involved in the regulation of nucleotide pools and magnesium transporters as a mechanism to cope with ribosomal stress caused by arsenic, which is exacerbated when pentavalent arsenic, which is a strong phosphate imitator, is present. 

### 4.4. Zinc

There was a stark difference observed between the two strains of *E. coli* tested regarding the changes in the zinc metalloproteomes due to arsenic treatment. The zinc metalloproteins were significantly decreased with As^III^ treatment and further decreased with As^V^ treatment in AW3110, but little to no difference in the zinc metalloproteome was observed in K-12 *E. coli* cells. Interestingly, arsenic treatment increased the total concentration of soluble zinc for K-12, and only the As^V^ treatment of AW3110 reduced the soluble zinc concentration (Figure 4d). An explanation for this may come from zinc studies in other organisms. Zinc has been shown to regulate the antioxidant defense system in rats in response to As^III^ treatment [36]. Zinc was also reported to have antagonist effects against arsenic in preterm births of pregnant women in Bangladesh [37]. Transcriptomic analysis by Rawle et al. showed the increased transcription of an ABC transporter, zinc-containing alcohol dehydrogenases, and a TraR/DksA C4-type zinc finger protein [13]. Interestingly, As^III^ has been shown to displace zinc in zinc-finger proteins containing three or more cysteines [38]. High levels of As^III^ cause a scarcity of zinc in an arsenic-resistant *Paenibacillus taichungensis* isolated from a gold–copper mine [39]. *P. taichungensis* upregulates a zinc uptake system and a zinc metallochaperone, which have been hypothesized to be a coping mechanism against oxidative stress. Our data support an emerging notion in the literature that zinc plays an important role in the detoxification of arsenic. Our data further suggest that the *arsRBC* operon may regulate the zinc metalloproteome.

### 4.5. Iron

In the same arsenic-resistant *P. taichungensis* study, the downregulation of genes involved in iron uptake was observed following As^III^ exposure, which was attributed to be part of an oxidative stress response mechanism [39]. The authors supported this interpretation of the data by pointing to iron as a major source of biological reactive oxygen species and the cells’ needs to limit the labile iron pool to avoid oxidative damage from both iron and arsenic reactive oxygen species generation. Our data on *E. coli* do not support this hypothesis because the total soluble iron increased in K-12 following As^III^ and As^V^ treatment. K-12 cell treatment with As^III^, however, caused a significant decrease in iron-containing proteins, while As^V^ treatment led to an increase in high molecular weight proteins or protein complexes, with no significant difference in iron-protein abundance (Figure 2). A similar shift is observed in the absence of the *arsRBC* operon upon As^V^ treatment of AW3110 *E. coli* cells. Unlike in K-12, both As^III^ and As^V^ treatment caused a significant decrease in iron-bound proteins in AW3110 (Figure 3). The presence of the *arsR* genes in the gene knockout study by Rawle et al. showed increased and decreased expression of gene transcripts coding for iron transporters, depending on which *arsR* gene was present. Our data demonstrate that there are detectable changes in the iron proteome following arsenic treatments. Additionally, the data support involvement of the *arsRBC* operon in iron homeostasis, as put forth by Rawle et al.

### 4.6. Protein Cofactor and Redox Status

As a final analysis of intracellular metalloproteome status, we monitored three wavelengths to probe protein cofactor changes in K-12 and AW3110 *E. coli* as a function of arsenic treatment. The wavelengths monitored were chosen because absorption at 360 nm and 460 nm will detect oxidized flavin [20], and monitoring at 405 nm can detect heme [21]; however, this method is non-specific, and other molecules can influence the monitored wavelengths [40]. Despite this non-specificity, there are clear differences between the two strains of *E. coli*. In K-12, the biggest changes appear to be from As^V^ treatment. These changes were mostly reflected in spectral intensity changes, while shifts in the elution time associated with these signals were minimal (Figure 5a). In AW3110, however, As^III^ treatment induced a shift in absorbance to lower molecular weight protein/protein complexes for all wavelengths, while As^V^ treatment mainly reduced the signal intensity over the entire elution window (Figure 5b). It is difficult to definitively say whether the observed changes are due to changing redox status, but the current literature shows that arsenic does influence the redox status of organisms. A study examining the long-term effect of As^III^ treatment in adult rats showed that As^III^ inhibits several intermediary enzymes of heme metabolism, as well as alters the intra- and extracellular porphyrin content [41]. Beyond heme, other changes of cellular redox status due to arsenic are expected. The ArsC protein in the *E. coli* K-12 strain, for example, uses reduced glutathione and glutaredoxin as electron donors [42]. Glutathione is important because it is involved in Zn^II^ and Cu^II^ homeostasis and toxicity resistance in *E. coli* [43]. As^III^ exposure has been shown to lower the ratio of reduced to oxidized glutathione, enhance glutathione transferase activity, as well as increase the oxidation of protein thiols in *Rhodotorula mucilaginous* [44]. This, together with the conserved zinc metalloproteome of K-12 *E. coli* cells following arsenic treatment, suggests that there may be an interplay between the *arsRBC* operon, metal homeostasis, and protein-bound cofactor abundance. 

## 5. Concluding Remarks

To the best of our knowledge, this is the first SEC-ICPMS analysis of *E. coli* bacterial metalloproteome in response to arsenic stress. The data clearly demonstrate that arsenic treatment causes global changes in the metalloproteome of *E. coli*, and that these changes differ between the two strains of *E. coli* (K-12 and AW3110) when the cells are under similar levels of metal-induced stress. Our work further supports the hypothesis postulated by Rawle et al. that the *ars* operon is involved in the widespread regulation in bacteria, and that arsenic detoxification is a complex process involving more than just the three proteins encoded by the *arsRBC* gene locus. This study sets the stage for future experiments investigating the role of individual *arsR, arsB,* and *arsC* genes regarding protein expression and the metalloproteome.

## Data Availability

The raw data generated during the current study are available from the corresponding author upon request.

## Supplementary Materials

The following supporting information can be downloaded at: https://www.mdpi.com/article/10.3390/microorganisms11020382/s1.
