# Supplementary material for: Arsenic Exposure Causes Global Changes in the Metalloproteome of Escherichia coli
Source: Microorganisms. 2023 Feb 2;11(2):382. doi: 10.3390/microorganisms11020382 (PMC9965246; doi:10.3390/microorganisms11020382)
Supplement: Supplementary file 1 [file microorganisms-11-00382-s001.zip › SuppFigures_Final_v2.pdf]

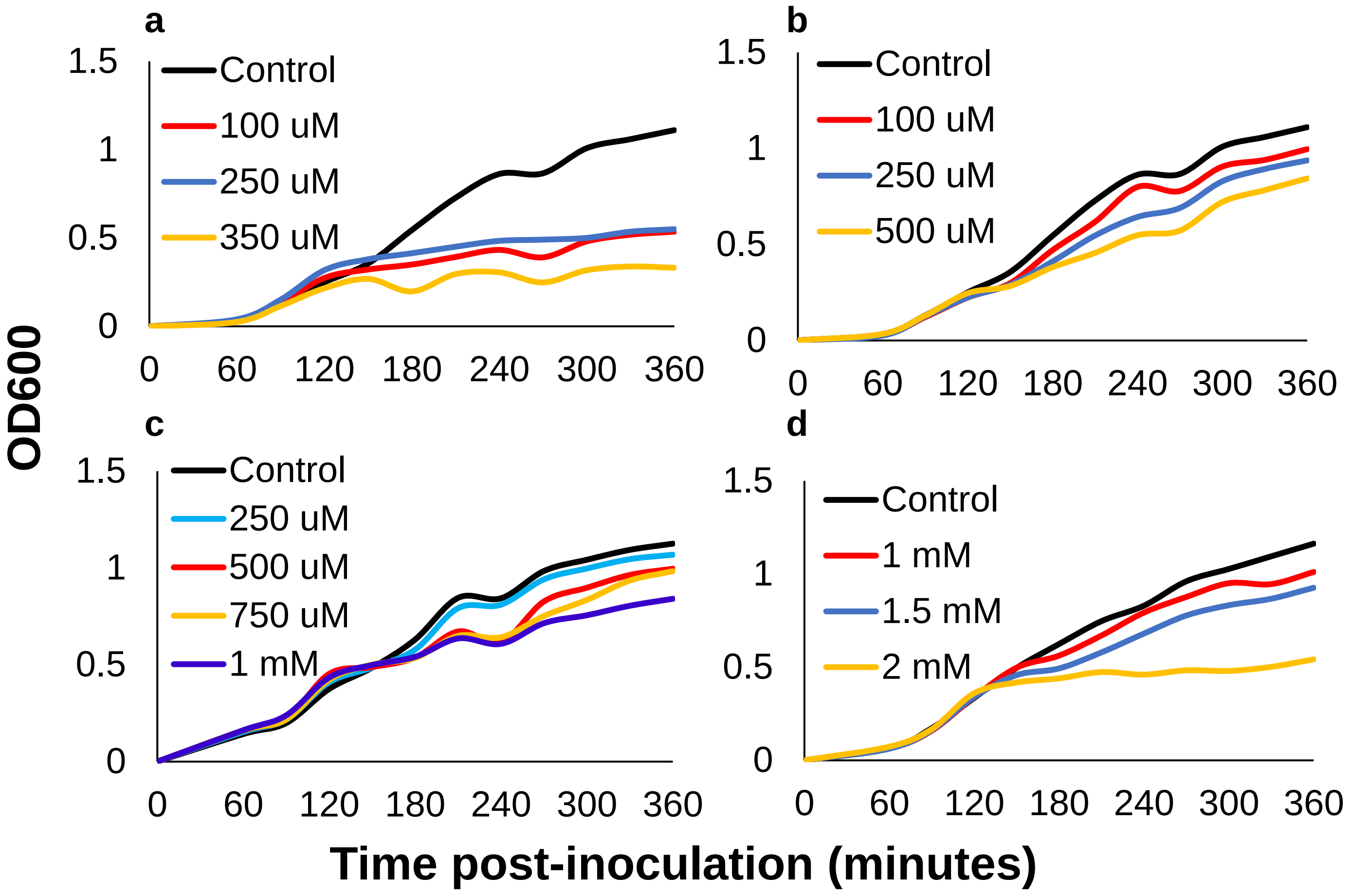

Supplemental Figure S1: Representative growth curves. AW3110 (a and b) and K-12 (c and d) were cultured with increasing concentrations of As^{III} (a and c) or As^{V} (b and d). Arsenic was added 120 minutes post-inoculation.

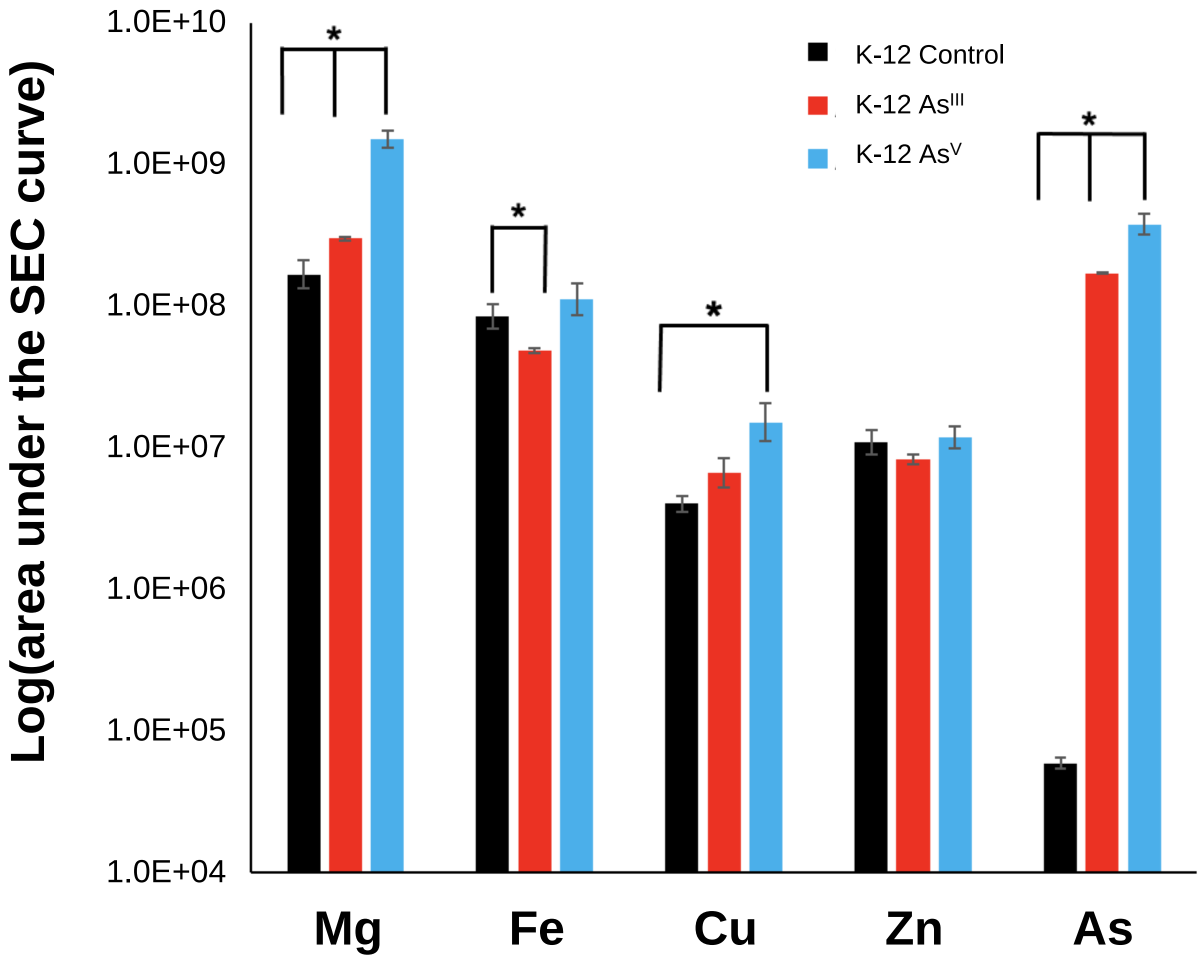

Supplemental Figure S2: K-12 SEC-ICPMS metal profile integrations. The area under the curve for metal profiles for protein-bound copper, magnesium, arsenic, iron, and zinc from SEC-ICP-MS analysis. As^{V} treatment is in cyan, As^{III} treatment is in red, and control is in black. Error bars are +/- 1 standard deviation. * *indicates significant differences (p < 0.05) determined by student's T-test.*

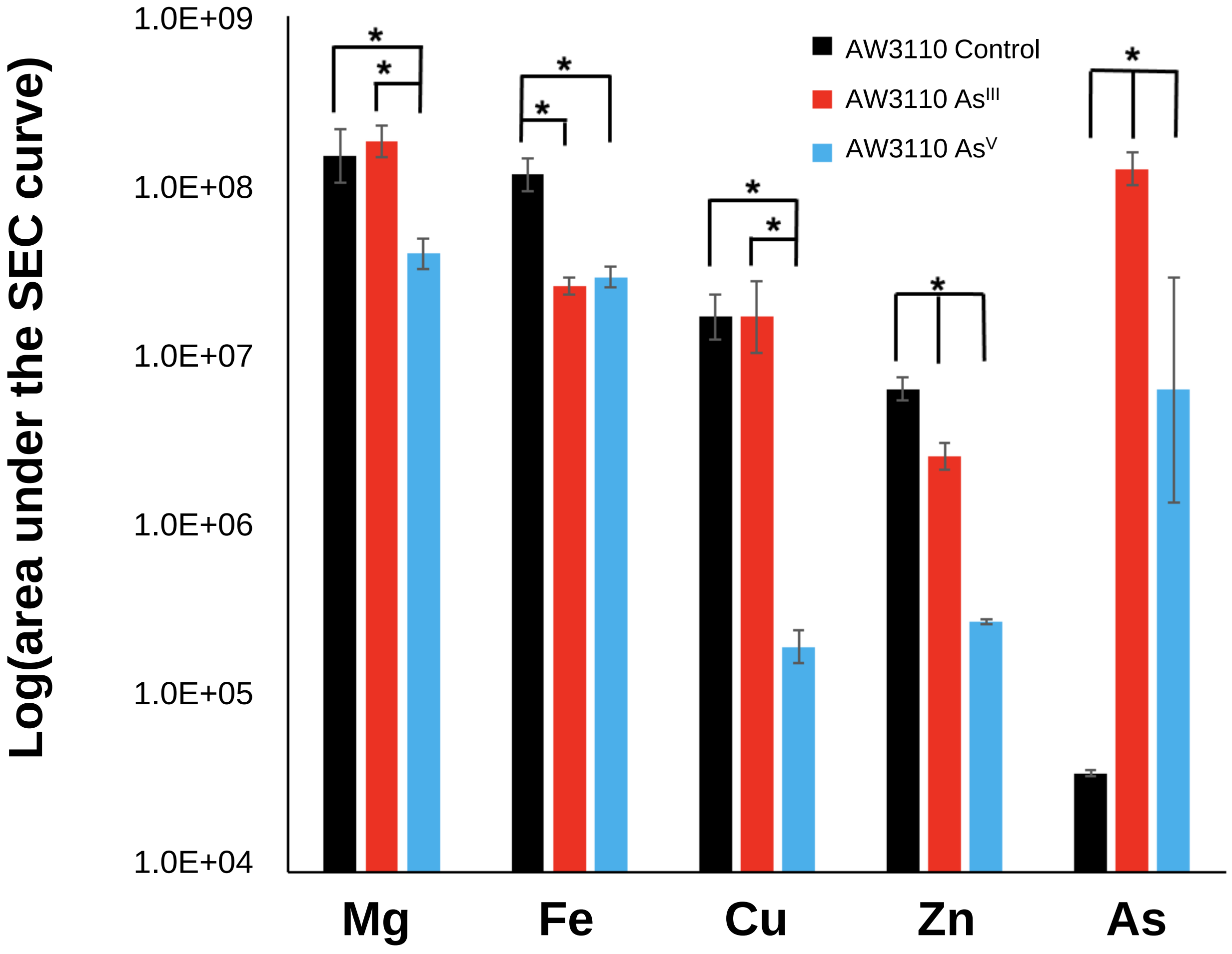

Supplemental Figure S3: AW3110 SEC-ICPMS metal profile integrations. The area under the metalloprotein profile curves for copper, magnesium, arsenic, iron, and zinc from SEC-ICP-MS analysis. As^{V} treatment is in cyan, As^{III} treatment is in red, and control is in black. Error bars are +/- 1 standard deviation. ** indicates significant differences (p < 0.05) determined by student's T-test.*

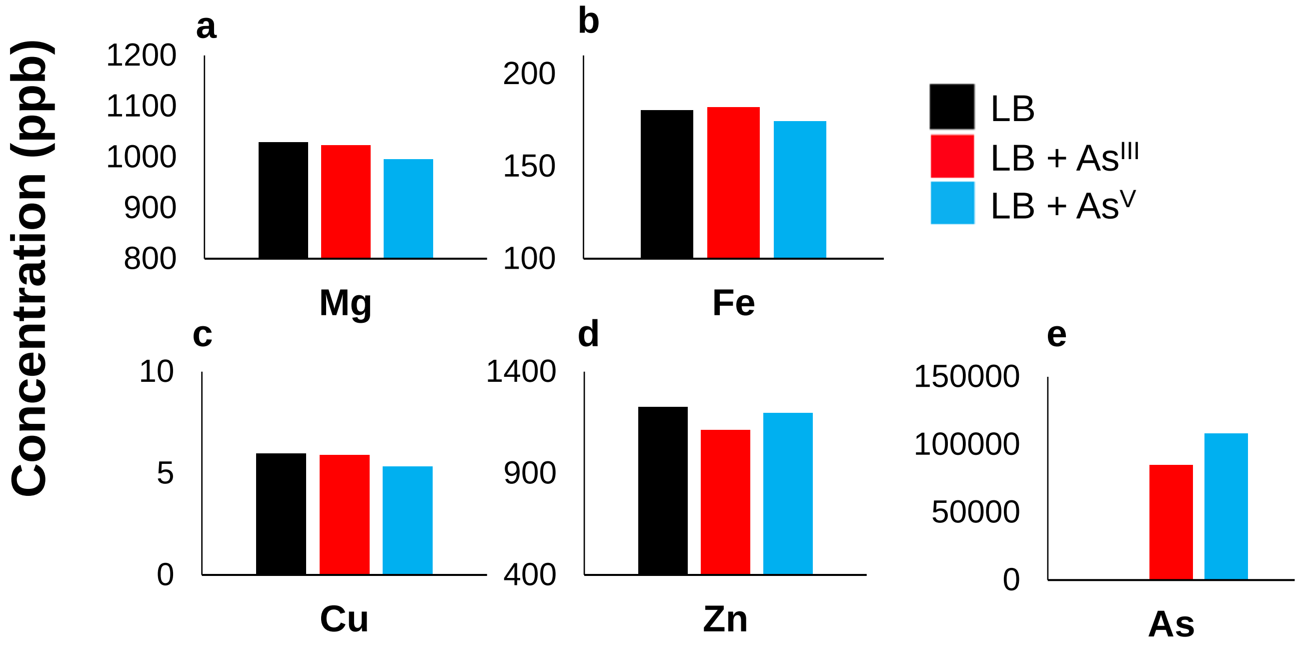

Supplemental Figure S4: Metal concentration in media. LB media without arsenic supplementation (black), with As^{III} (red), and with As^{V} (cyan) was analyzed via ICP-MS for Mg (a), Fe (b), Cu (c), Zn (d), and As (e).

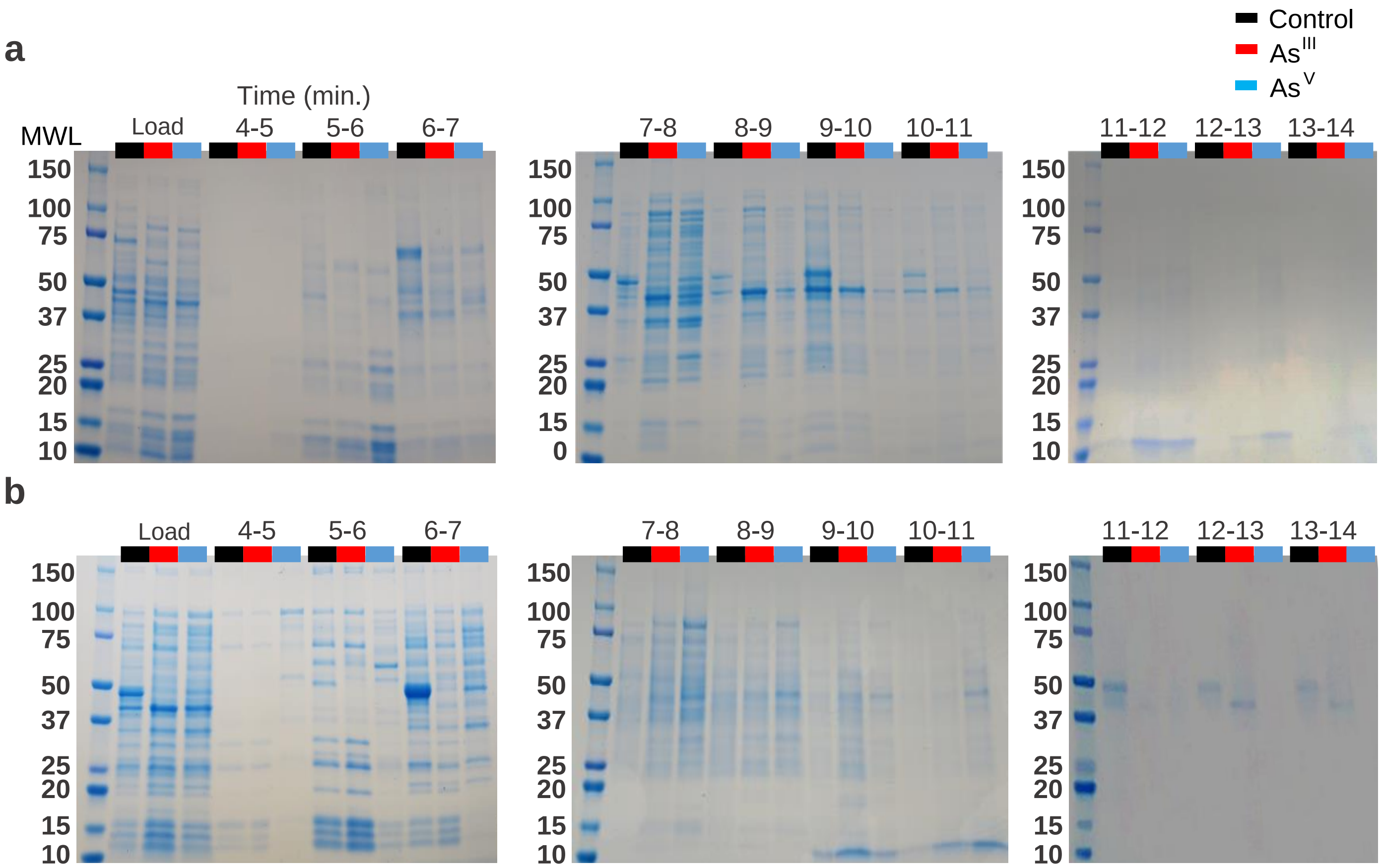

Supplemental Figure S5: SDS-PAGE analysis of SEC fractionations. Fractions for K-12 (a) and AW3110 (b) were collected during SEC-UV analysis and analyzed by SDS-PAGE. 1-min Fractions began at minute 4 post-injection (fraction 1) and ended at minute 15 (fraction 10).
